# Development of a CRISPR-Cas12a based assay for the detection of swine enteric coronaviruses in pig herds in China

**DOI:** 10.1007/s44307-024-00015-x

**Published:** 2024-02-27

**Authors:** Yongbo Xia, Yue Li, Yihong He, Xiaowei Wang, Wenjing Qiu, Xiaoyuan Diao, Yunfei Li, Junfeng Gao, Hanqin Shen, Chunyi Xue, Yongchang Cao, Peng Li, Zhichao Xu

**Affiliations:** 1 State Key Laboratory of Biocontrol, School of Life Sciences, Sun Yat-Sen University, Guangzhou, 510006 China; 2 Department of Immunology and Genomic Medicine, National Jewish Health, Denver, CO 80206 USA; 3 Yunfu Branch, Guangdong Laboratory for Lingnan Modern Agriculture, Yunfu, 527400 China; 4 Veterinary Diagnostic and Production Animal Medicine, Iowa State University, Ames, 50010 USA

**Keywords:** Swine enteric coronaviruses, CRISPR-Cas12a, Nucleocapsid gene, Detection assay

## Abstract

Porcine epidemic diarrhea virus (PEDV), Transmissible gastroenteritis virus (TGEV), Porcine deltacoronavirus (PDCoV) and Swine acute diarrhea syndrome coronavirus (SADS-CoV) rank among the most frequently encountered swine enteric coronaviruses (SECoVs), leading to substantial economic losses to the swine industry. The availability of a rapid and highly sensitive detection method proves beneficial for the monitoring and surveillance of SECoVs. Based on the *N* genes of four distinct SECoVs, a novel detection method was developed in this study by combining recombinant enzyme polymerase isothermal amplification (RPA) with clustered regularly interspaced short palindromic repeats (CRISPR)-associated proteins (Cas) 12a. Results showed that the cut-off value of CRISPR-Cas12a assay for SADS-CoV, PEDV, PDCoV and TGEV was 2.19 × 10^4^ Relative Fluorescence Units (RFU), 1.57 × 10^4^ RFU, 3.07 × 10^4^ RFU and 1.64 × 10^4^ RFU, respectively. The coefficient of variation (CV) of within and between runs by CRISPR-Cas12a assay for 6 clinical diarrhea samples were both less than 10%. The CRISPR-Cas12a assay demonstrated high specificity for TGEV, PEDV, PDCoV, and SADS-CoV with no cross-reactivity to other common swine viruses. This method also exhibited a low limit of detection of 2 copies for each virus. Additionally, the results demonstrated a perfect agreement (100%) between the CRISPR-Cas12a assay and the RT-qPCR assay. Finally, a total of 494 pig samples from the field tested by CRISPR-Cas12a assay showed that positive rate for SADS-CoV, TGEV, PDCoV and PEDV was 0, 0, 1.2% and 48.6%, respectively. The results suggested the great potential of CRISPR-Cas12a assay to detect SECoVs in the field.

## Introduction

Coronaviruses (CoVs), including SARS-CoV-2, are a substantial concern for human and animal health (Decaro and Lorusso 2020). CoVs are found in a wide variety of animals (Xu et al. 2018; Haake et al. 2020). They belong to the subfamily *Coronavirinae* in the family *Coronaviridae* within the order *Nidovirales* (Xu et al. 2018). Transmissible gastroenteritis virus (TGEV), Porcine epidemic diarrhea virus (PEDV), Porcine deltacoronavirus (PDCoV), and Swine Acute Diarrhea Syndrome Coronavirus (SADS-CoV) belong to the group of swine enteric coronaviruses (SECoVs), which can cause gastrointestinal-related diseases in pigs (Yan et al. 2022; Yang et al. 2020). SECoVs are a group of viruses that have an envelope and a positive-sense, single-stranded RNA genome. The size of their genome is typically around 27–32 kilobases (Brian and Baric 2005). SECoVs genome encode a variety of non-structural proteins as well as four structural proteins. These structural proteins are spike (S), membrane (M), envelope (E), and nucleocapsid (N) proteins (Chen et al. 2020). Among them, the N protein is primarily responsible for binding to the RNA genome and plays a crucial role in the assembly and release of the virus (Chen et al. 2020; Lu et al. 2020). Over the course of evolution, *N* genes remains one of the most conserved genes, while *S* genes often undergo rapid mutation (Domingo et al. 2021; Zhai et al. 2023). Therefore, many studies have selected *N* gene to develop detection method for the SECoVs.

SECoVs present clinical symptoms that include severe watery diarrhea and subsequent dehydration in pigs of all ages. Suckling piglets, in particular, have a high mortality rate, resulting in significant economic losses to the pig industry (Wen et al. 2018; Xia et al. 2018; Xu et al. 2019; Zhang 2016). In addition, some SECoV demonstrated high potential to spread across species, posing a threat to human public health (Cui et al. 2019; Edwards et al. 2020; Lednicky et al. 2021; Wang et al. 2018; Yang et al. 2019). Thus, monitoring of SECoVs is of great significance to pig industry and public health.

Rapid and sensitive tests are key to the virus monitoring. Currently, a variety of techniques have been developed and employed to detect SECoV infection including real-time fluorescence quantitative PCR (RT-qPCR), virus neutralization assay, reverse transcription loop-mediated isothermal amplification, reverse transcription recombinase polymerase amplification and enzyme-linked immunosorbent assay (Li and Ren 2011; Liu and Wang 2021; Sozzi et al. 2010). However, these methods have some drawbacks, such as being time-consuming, having high false-positive rates, and requiring expensive instruments. Thus, new detecting method are needed to offset disadvantages of current methods. Clustered regularly interspaced short palindromic repeats (CRISPR) and CRISPR associated proteins (Cas), also known as the CRISPR-Cas system, is an adaptive immune system found in archaea and bacteria. This remarkable system functions by efficiently identifying and degrading foreign nucleic acids to provide protection against invading genetic material (Manghwar et al. 2019; Marraffini and Sontheimer 2010). Cas12a, also known as Cpf1, is a V-type CRISPR-associated protein that specifically cleaves and degrades double-stranded DNA sequences under the guidance of crRNA (Chen et al. 2018). In addition to this targeted cleavage, Cas12a activates a bypass cleavage pathway upon binding to specific target DNA, leading to non-specific cleavage of adjacent non-target single-stranded DNA (Paul and Montoya 2020; Zetsche et al. 2015). The utilization of Cas12a's *trans-*cleavage activity on single-stranded DNA for nucleic acid detection has been reported in several studies (Li et al. 2018; Chen et al. 2018; Gootenberg et al. 2018). To enhance the sensitivity of detection, it is typically necessary to perform pre-amplification of nucleic acid using either an isothermal or PCR method. This step helps to increase the concentration of target genetic material, thereby improving the overall detection sensitivity (Xiong et al. 2020). The combination of Cas12a with recombinase polymerase amplification (RPA) technology has been employed to develop as a diagnostic assay for various pathogens, including SARS-CoV-2 and ASFV (Broughton et al. 2020; Wang et al. 2020).

Thus, the aim of this research was to establish a SECoVs detection method using a CRISPR-Cas12a-based assay.

## Materials and methods

### Design and preparation of crRNAs

Three high-scoring crRNAs target to the SECoVs *N* genes were designed from the crRNA design website (https://cctop.cos.uni-heidelberg.de:8043/). To create crRNA-F, the T7 promoter sequence (TAATACGACTCACTATAGGG) was appended to the 5' end of the target sequence. crRNA-R was generated by taking the reverse complement of the target sequence (Table 1). FAM-TTTTT-BHQ1 was synthesized by Sangon Biotech Co., Ltd (Shanghai, China). All crRNA-F/R were synthesized by Sangon Biotech Co., Ltd (Shanghai, China), then annealed to a double-stranded DNA using NEBuffer™ r 2.1 (NEB, USA). Subsequently, DNA fragments were purified using gel extraction and they were transcribed into crRNA using the Transcription T7 High Yield Transcription Kit (Thermo, USA). Finally, crRNAs were purified using RNA Clean & ConcentratorTM-5 (Zymo Research, USA) and stored at -80℃ until use.

**Table 1:** crRNAs and reporters used for CRISPR-Cas12a-based SECoVs detection

Primer name	Primer sequence (5'-3')
PEDV_crRNA1_F	GAAATTAATACGACTCACTATAGGGGTAATTTCTACTAAGTGTAGATAATGCATCCACCTGTGAAACAAG
PEDV_crRNA1_R	CTTGTTTCACAGGTGGATGCATTATCTACACTTAGTAGAAATTACCCCTATAGTGAGTCGTATTAATTTC
PEDV_crRNA2_F	GAAATTAATACGACTCACTATAGGGGTAATTTCTACTAAGTGTAGATGCACCAAATGTTGCAGCATTGCT
PEDV_crRNA2_R	AGCAATGCTGCAACATTTGGTGCATCTACACTTAGTAGAAATTACCCCTATAGTGAGTCGTATTAATTTC
PEDV_crRNA3_F	GAAATTAATACGACTCACTATAGGGGTAATTTCTACTAAGTGTAGATGAATGGGACACAGCTGTTGATGG
PEDV_crRNA3_R	CCATCAACAGCTGTGTCCCATTCATCTACACTTAGTAGAAATTACCCCTATAGTGAGTCGTATTAATTTC
SADS-CoV_crRNA1_F	GAAATTAATACGACTCACTATAGGGGTAATTTCTACTAAGTGTAGATGAGTAAGCCGAGACTTGCTCAAG
SADS-CoV_crRNA1_R	CTTGAGCAAGTCTCGGCTTACTCATCTACACTTAGTAGAAATTACCCCTATAGTGAGTCGTATTAATTTC
SADS-CoV_crRNA2_F	GAAATTAATACGACTCACTATAGGGGTAATTTCTACTAAGTGTAGATACTGCAGCAACAATGTCAACAGA
SADS-CoV_crRNA2_R	TCTGTTGACATTGTTGCTGCAGTATCTACACTTAGTAGAAATTACCCCTATAGTGAGTCGTATTAATTTC
SADS-CoV_crRNA3_F	GAAATTAATACGACTCACTATAGGGGTAATTTCTACTAAGTGTAGATGGGGCAGGTGCAGGTGAGACAGC
SADS-CoV_crRNA3_R	GCTGTCTCACCTGCACCTGCCCCATCTACACTTAGTAGAAATTACCCCTATAGTGAGTCGTATTAATTTC
PDCoV_crRNA1_F	GAAATTAATACGACTCACTATAGGGGTAATTTCTACTAAGTGTAGATGTGGCAATGGAGTTCCGCTTAAC
PDCoV_crRNA1_R	GTTAAGCGGAACTCCATTGCCACATCTACACTTAGTAGAAATTACCCCTATAGTGAGTCGTATTAATTTC
PDCoV_crRNA2_F	GAAATTAATACGACTCACTATAGGGGTAATTTCTACTAAGTGTAGATCTCTGGGACCTGTGCCAGTATAA
PDCoV_crRNA2_R	TTATACTGGCACAGGTCCCAGAGATCTACACTTAGTAGAAATTACCCCTATAGTGAGTCGTATTAATTTC
PDCoV_crRNA3_F	GAAATTAATACGACTCACTATAGGGGTAATTTCTACTAAGTGTAGATGCTGGCCACCTTGAGAGCAACTT
PDCoV_crRNA3_R	AAGTTGCTCTCAAGGTGGCCAGCATCTACACTTAGTAGAAATTACCCCTATAGTGAGTCGTATTAATTTC
TGEV_crRNA1_F	GAAATTAATACGACTCACTATAGGGGTAATTTCTACTAAGTGTAGATGTGACACTGACCTCGTTGCCAAT
TGEV_crRNA1_R	ATTGGCAACGAGGTCAGTGTCACATCTACACTTAGTAGAAATTACCCCTATAGTGAGTCGTATTAATTTC
TGEV_crRNA2_F	GAAATTAATACGACTCACTATAGGGGTAATTTCTACTAAGTGTAGATAGTGCGGCAAGAACAGCTTGTTC
TGEV_crRNA2_R	GAACAAGCTGTTCTTGCCGCACTATCTACACTTAGTAGAAATTACCCCTATAGTGAGTCGTATTAATTTC
TGEV_crRNA3_F	GAAATTAATACGACTCACTATAGGGGTAATTTCTACTAAGTGTAGATGAACGAGAGCGTTGCTGTTGTTT
TGEV_crRNA3_R	AAACAACAGCAACGCTCTCGTTCATCTACACTTAGTAGAAATTACCCCTATAGTGAGTCGTATTAATTTC
ssDNA reporter	6-FAM-TTATT-BHQ1

### Primers design and RT-RPA reactions

RT-RPA primers targeting the *N* genes of SECoVs were designed with reference to the protocol of Twist-Dx (Cambridge, United Kingdom) based on published sequences [SADS-CoV (GenBank ID: MH697599.1); PEDV (GenBank ID: KM089829.1); PDCoV (GenBank ID: MH715491.1); TGEV (GenBank ID: KX499468.1)]. Pimer pairs were initially screened by pairing a forward primer with various reverse primers, then the optimal reverse primer was selected to screen all the forward primers. The selected primer pairs were used in CRISPR-Cas 12a based assay (Table 2). RT-RPA reactions were conducted according to the manufacturer's instructions using a kit (AmpFuture, Weifang, China). Briefly, the RT-RPA was performed in a 50-μL volume containing RT-RPA enzymes, 2 μL of RNA, and 0.4 μM of each primer. All reactions were incubated at 42˚C for 30 min.

**Table 2:** Primers used for RPA-only detection

Primer name	Primer sequence (5'-3')
SADS-CoV_RPA_F1	CTTCCGACACAAGCTGCACTAGCCTTTGGTAG
SADS-CoV_RPA_R1	ACTGGGGCATCAGCATTTAGGTCTTGTTGAGA
SADS-CoV_RPA_F2	TTCCGACACAAGCTGCACTAGCCTTTGGTAG
SADS-CoV_RPA_R2	ACACTGGGGCATCAGCATTTAGGTCTTGTTGA
SADS-CoV_RPA_F3	ACACAAGCTGCACTAGCCTTTGGTAGTGAAAT
SADS-CoV_RPA_R3	TGAACACTGGGGCATCAGCATTTAGGTCTTGT
SADS-CoV_RPA_F4	CTTCCGACACAAGCTGCACTAGCCTTTGGTA
SADS-CoV_RPA_R4	CACTGGGGCATCAGCATTTAGGTCTTGTTGAG
SADS-CoV_RPA_F5	CACAAGCTGCACTAGCCTTTGGTAGTGAAATC
SADS-CoV_RPA_R5	GAGTGAACACTGGGGCATCAGCATTTAGGTCT
PEDV_RPA_F1	TGGTAATGTAAAACCCCAGAGAAAGAAGGAAA
PEDV_RPA_R1	ATTTCCTGTATCGAAGATCTCGTTGATAATTT
PEDV_RPA_F2	AACTGGTAATGTAAAACCCCAGAGAAAGAAGG
PEDV_RPA_R2	AATTTCCTGTATCGAAGATCTCGTTGATAATTT
PEDV_RPA_F3	TGCATTTAAAACTGGTAATGTAAAACCCCAGA
PEDV_RPA_R3	TTTCCTGTATCGAAGATCTCGTTGATAATTT
PEDV_RPA_F4	TGTAAAACCCCAGAGAAAGAAGGAAAAGAAGA
PEDV_RPA_R4	TAATTTCCTGTATCGAAGATCTCGTTGATAATTT
PEDV_RPA_F5	AAACCCCAGAGAAAGAAGGAAAAGAAGAACAA
PEDV_RPA_R5	TTAATTTCCTGTATCGAAGATCTCGTTGATAATTT
PDCoV_RPA_F1	CAGACACTGAGAAGACGGGTATGGCTGATCCT
PDCoV_RPA_R1	GCGCATCCTTAAGTCTCTCATAGTCAGGAGAA
PDCoV_RPA_F2	AGACACTGAGAAGACGGGTATGGCTGATCCTC
PDCoV_RPA_R2	GCGCATCCTTAAGTCTCTCATAGTCAGGAGAACCC
PDCoV_RPA_F3	ACACTGAGAAGACGGGTATGGCTGATCCTCG
PDCoV_RPA_R3	TGAGCGCATCCTTAAGTCTCTCATAGTCAGGA
PDCoV_RPA_F4	GAAGACGGGTATGGCTGATCCTCGCATCATGG
PDCoV_RPA_R4	TGAGCGCATCCTTAAGTCTCTCATAGTCAGGAG
PDCoV_RPA_F5	GGGTATGGCTGATCCTCGCATCATGGCTCTA
PDCoV_RPA_R5	TTGAGCGCATCCTTAAGTCTCTCATAGTCAGGA
TGEV_RPA_F1	AGGCAGGCAACAATTCAATAACAAGAAGGATG
TGEV_RPA_R1	ACCTGCAGTTCTCTTCCAGGTGTGTTTGTTTT
TGEV_RPA_F2	GGCAGGCAACAATTCAATAACAAGAAGGATGA
TGEV_RPA_R2	CCTTTACCTGCAGTTCTCTTCCAGGTGTGTTT
TGEV_RPA_F3	GAACAAGCTGTTCTTGCCGCACTTAAAAAGTT
TGEV_RPA_R3	GTCACATCACCTTTACCTGCAGTTCTCTTCCA
TGEV_RPA_F4	AACAAGCTGTTCTTGCCGCACTTAAAAAGTTA
TGEV_RPA_R4	TGTCACATCACCTTTACCTGCAGTTCTCTTCC
TGEV_RPA_F5	GAGGCAGGCAACAATTCAATAACAAGAAGGAT
TGEV_RPA_R5	TACCTGCAGTTCTCTTCCAGGTGTGTTTGTTT

### Extraction of viral genomic nucleic acids and CRISPR-Cas12a detection reactions

To facilitate viral RNA extraction, the intestinal contents were diluted with sterile 1 × phosphate buffer saline (PBS) at a pH of 7.4. Following the dilution, the supernatants were collected by centrifuging the dilution at 5000 × *g* for 5 min at 4°C. Total RNA was extracted from the supernatants using a RNeasy kit (Magen, China) following the manufacturer's instructions, and subsequently treated with DNase I to remove any contaminating DNA. The RT-RPA method was employed to amplify complementary DNA (cDNA) from the extracted RNA. The CRISPR-Cas12a reaction was performed in a 20-μL volume containing 2 μL cDNA, 2 μL 10 × NEBuffer 2.1 (NEB, Ipswich, USA), 50 nM Cas12a (NEB, Ipswich, USA), 100 nM crRNA, and 250 nM ssDNA probe reporter. The reactions were incubated in a fluorescence plate reader (BioTek Synergy H1, USA) and incubated at 37°C for 90 min. Fluorescent signals were collected every 15 min during the incubation period (ssDNA FQ substrates = λ ex: 485 nm; λ em: 535 nm).

### Determination of the cut-off value of CRISPR-Cas12a based assay

The cut-off value of CRISPR-Cas12a based assay was determined as previously described with some modifications (Li et al. 2015). In brief, to determine the cut-off value, negative pig diarrheic samples, as verified by RT-qPCR, were tested using the CRISPR-Cas 12a based assay. Each sample was subjected to three replicates, and the mean fluorescence values of all negative samples at 15 min, along with three standard deviations (SDs), were utilized as the cut-off value. Samples with fluorescence values equal to or higher than this threshold at 15 min were considered positive, while those below this cut-off were considered negative. This cut-off gives 99% confidence that all negative values will fall within the defined range. SECoVs were used as positive controls.

### Determination of repeatability of CRISPR-Cas12a based assay

Six pig diarrhea samples (three RT-qPCR-positive samples and three RT-qPCR-negative samples) were selected for repeatability assay as previously described with some modifications (Lin et al. 2018). To assess intra-assay reproducibility, each sample was subjected to three replicates on the same plate during a single occasion. For inter-assay reproducibility, each sample was tested in three replicates across different plates and on different occasions. The results were expressed as the coefficient of variation (CV), calculated by dividing the standard deviation (SD) by the mean fluorescence value of each group of samples. A CV value of within 10% was deemed to meet the repeatability requirement.

### Determination of sensitivity of CRISPR-Cas12a based assay

To determine the limit of detection of the CRISPR-Cas12a based assay, the recombinant plasmid pMD19-T-*N* of each virus was diluted in a gradient ranging from 2 × 10^7^ to 2 × 10^0^ copies/μL, Subsequently, 2 μL of each dilution was tested using the CRISPR-Cas12a based assay. The formula for calculating the RNA copy number is as follows: RNA copy number = (M × 6.022 × 10^23^)/(n × 1 × 10^9^ × 660), in which M represents the amount of DNA in nanograms, n is the length of the DNA in base pair, and the average weight of a base is assumed to be 330 Daltons.

### Cross-reactivity of CRISPR-Cas12a based assay to other swine major pathogens

To determine the cross-reactivity, the PEDV strain GDS01 (Hao et al. 2014), PDCoV strain CHN-GD-2016 (Xu et al. 2018), SADS-CoV strain GDS04 (Gong et al. 2017) and some other common swine viruses including TGEV, Porcine atypical pest virus (APPV), Porcine reproductive and respiratory syndrome virus (PRRSV), African swine fever virus (ASFV), Swine fever virus (CSFV), Pseudorabies virus (PRV) and Porcine circovirus type 2 (PCV2) [TGEV, APPV, PRRSV, ASFV, CSFV, PRV and PCV2 were kindly provided by Professor Lang Gong (South China Agricultural University, China)] and tested using the CRISPR-Cas12a based assay. Each sample underwent three replicates. Based on the previously mentioned criteria, the sample was classified as positive or negative depending on whether the mean fluorescence value exceeded the designated threshold.

### Real-time RT-PCR analysis

Viral RNA extraction was described as above. The specific primers for the *N* genes of SECoVs (Table 3) were designed with reference to the previous publication (Kim et al. 2007, Ma et al. 2015, Xu et al. 2019) and synthesized by Invitrogen Company (Shanghai, China). The real-time PCR assay was carried out with an Applied Biosystem 7500 Fast instrument (Life Technologies, USA). The PCR reaction system was performed in a 20-μL volume containing 10 μL 2 × One Step Buffer, 0.8 μL One Step Enzyme Mix, 0.4 μL 50 × ROX Reference Dye 1 (Yisheng, Shanghai, China), 200 nM of each gene-specific primer, 100 nM of probe, 2 μL RNA, and 5.8 μL RNase-free ddH_2_O. ddH_2_O was used as the template for negative control. The thermal cycling parameters were as follows: 50 ℃ for 20 min, pre-denaturation at 95 ℃ for 5 min, 40 cycles of 94 ℃ for 15 s, 60 ℃ for 30 s.

**Table 3:** Primers and probes used for RT-qPCR detection

Primer name	Primer sequence (5'-3')
PEDV-F	CGCAAAGACTGAACCCACTAATTT
PEDV-R	TTGCCTCTGTTGTTACTTGGAGAT
PEDV-probe	FAM-TGTTGCCATTGCCACGACTCCTGC-TAMRA
PDCoV-F	CGCTTAACTCCGCCATCAA
PDCoV-R	TCTGGTGTAACGCAGCCAGTA
PDCoV-probe	FAM-CCCGTTGAAAACC-TAMRA
TGEV-F	GCAGGTAAAGGTGATGTGACAA
TGEV-R	ACATTCAGCCAGTTGTGGGTAA
TGEV-probe	FAM-TGGCACTGCTGGGATTGGCAACGA-TAMRA
SADS-CoV-F	GCACTTTTATTACCTTGGTA
SADS-CoV-R	GTAGCAGGTTCTTTGTTAC
SADS-CoV-probe	FAM-TCCTCACGCAGATGCTCCTT-TAMRA

### Detection of SADS-CoV, PEDV, TGEV and PDCoV in field samples by CRISPR-Cas12a based assay

From December 2021 to March 2023, a total of 494 porcine diarrhea samples were collected from 55 large-scale pig farms located in 13 provinces and cities across China (Guangdong, Guangxi, Guizhou, Hainan, Hubei, Hunan, Jilin, Jiangsu, Liaoning, Neimenggu, Anhui, Shanxi, Chongqing), and stored at -80 ℃ until further testing. The CRISPR-Cas12a based assay was employed to test all samples. SADS-CoV, PEDV, PDCoV, TGEV and ddH_2_O were used as positive and negative controls, respectively.

### Statistical analysis

Standard errors and coefficient of variation were performed using Excel and GraphPad Prism software 5.0 (GraphPad, San Diego, CA, USA), respectively.

## Results

### Development of a CRISPR-Cas12a based assay for Swine enteric coronaviruses

**Fig. 1:**
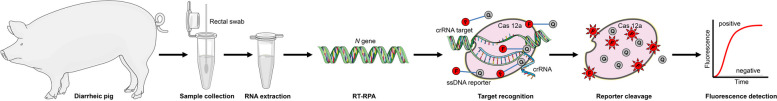
The flowchart of the CRISPR-Cas12a based assay for Swine enteric coronaviruses detection. Viral RNA was extracted from pig diarrheic samples, and the N target region amplified using RT-RPA. The amplicon was then mixed with Cas12a/crRNA, and a ternary complex formed when the target region was present. Finally binding to the target site resulted in the cleavage of the reporter ssDNA producing a signal. The signal could be detected by a fluorescent reader

A schematic CRISPR-Cas12a based assay for SECoVs is shown in Fig. 1. Viral RNA was extracted from pig diarrheic samples, and the *N* target region was amplified using RT-RPA. Subsequently, the resulting amplicon was combined with Cas12a/crRNA, leading to the formation of a ternary complex in the presence of the target region. Ultimately, when the CRISPR-Cas12a system bound to the designated region and the reporter single-stranded DNA (ssDNA) was cleaved, generating a detectable signal. The signal was then detected by a fluorescent reader. To ensure accurate targeting of the *N* gene, three high-scoring crRNAs were designed and synthesized for each virus, followed by a screening process. In the CRISPR-Cas12a assay established in this study, 500 ng RNA from each virus was used as a template. Three crRNAs were subjected to annealing, in vitro transcription and purification processes, from which crRNA with optimal performance were selected. The reaction lasted a total of 90 min, with the fluorescence values being measured and recorded every 15 min.

**Fig. 2:**
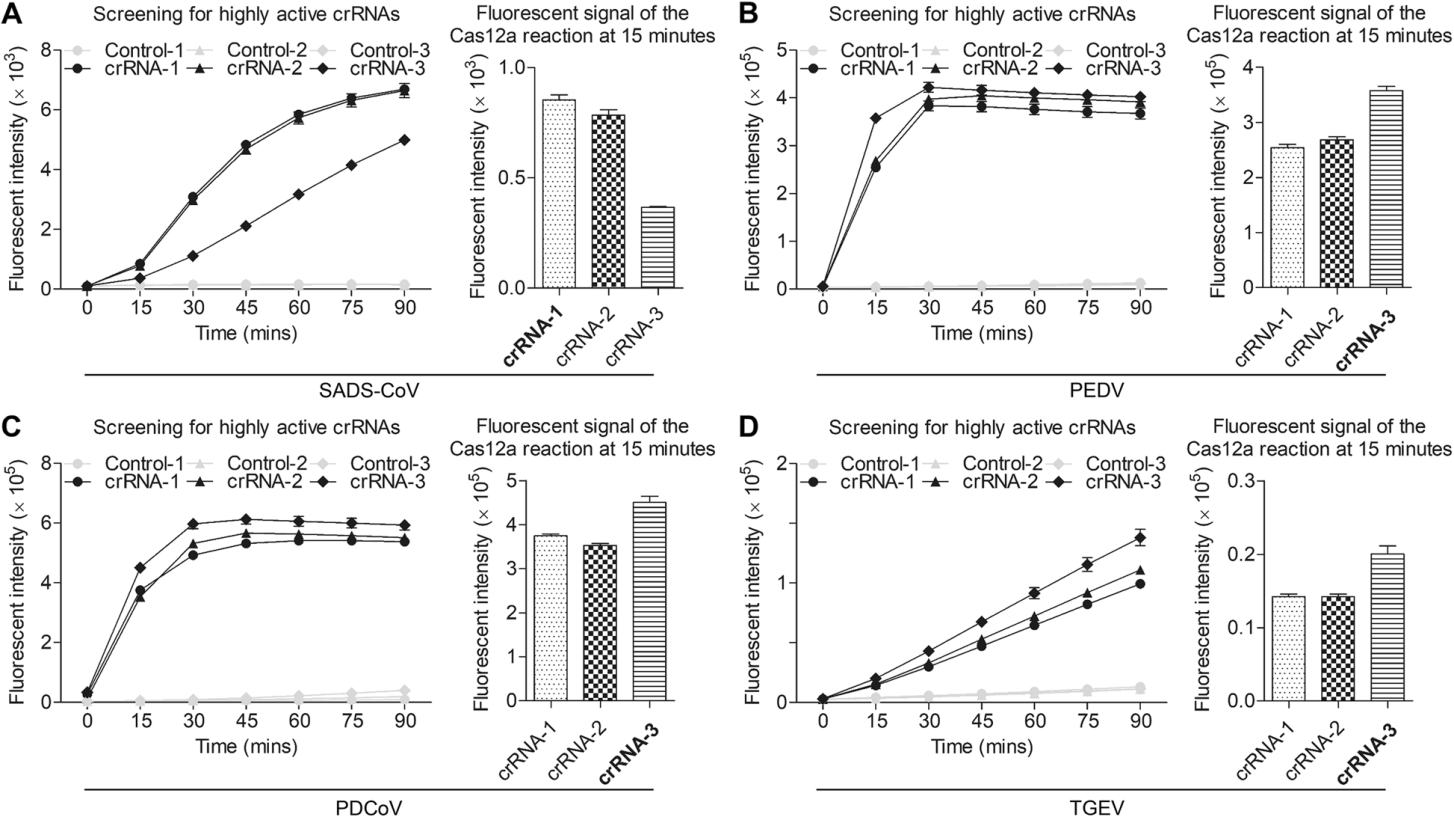
Screening for highly active crRNAs by CRISPR-Cas12a reaction. A total of 3 crRNAs targeting the *N* gene were designed, virus was used to test crRNA activity by RT-RPA-CRISPR-Cas 12a reaction. **A** Screening for SADS-CoV crRNA. **B** Screening for PEDV crRNA. **C** Screening for PDCoV crRNA. **D** Screening for TGEV crRNA. The line chart shows the change of fluorescence intensity of Cas12a reaction, the histogram shows the fluorescence intensity at 15 min, the black bold indicates the best crRNA (*n* = 3), and the data represents mean ± SD

**Fig. 3:**
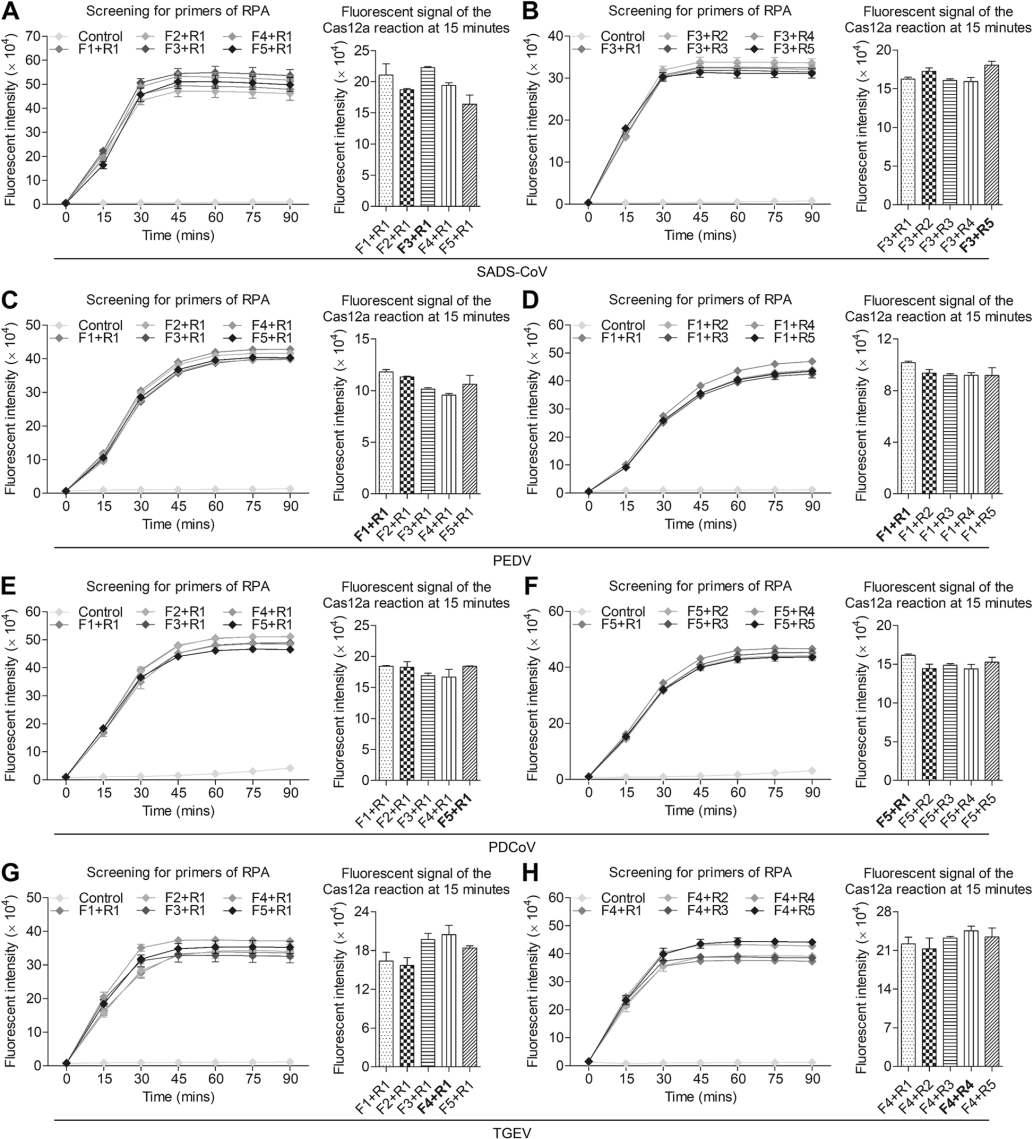
Screening for optimal primers by CRISPR-Cas12a reaction. Primers for *N* were screened using a single forward or reverse primer against the corresponding reverse or forward primers by RT-RPA-CRISPR-Cas 12a reaction, selecting the best performing primer and then using it to perform second round screening to determine the primer pairs with the best performance. **A, B** Screening for the optimal RT-RPA primers for SADS-CoV. **C, D** Screening for the optimal RT-RPA primers for PEDV. **E, F** Screening for the optimal RT-RPA primers for PDCoV. **G, H** Screening for the optimal RT-RPA primers for TGEV. The line chart shows the change of fluorescence intensity in Cas12a reaction, the histogram shows the fluorescence intensity at 15 min, the black bold indicates the best primer combination (*n* = 3), and the data represents mean ± SD

As shown in Fig. 2A, the fluorescence value increased by time and finally reached a plateau and the fluorescence intensity value of SADS-CoV crRNA-1 was higher than that of the other two crRNAs at 15 min. Thus crRNA-1 was chosen as the optimal crRNA. Similarly, the optimal crRNA for PEDV, PDCoV, TGEV were all crRNA-3 (Fig. 2B-D). As the sequences of oligonucleotides are crucial to ensure the specificity of a RT-RPA assay, five pairs of primers were designed, synthesized and further screened. As shown in Fig. 3A, B, to screen the forward primers (F1 to F5) of SADS-CoV, a fixed reverse primer (R1) was employed. After 15 min, the best performing forward primer (F3) was selected. This chosen forward primer (F3) was then utilized to screen all the available reverse primers (R1 to R5). Through this screening process, the primer pair demonstrating the optimal performance was identified and subsequently used in the subsequent experiments. In this case, the primer pair (F3 R5) displayed the optimal results. The same procedure was employed to screen primers for other SECoVs, and the primer set of PEDV F1 + R1, PDCoV F5 + R1, TGEV F4 + R4 demonstrate optimal performance at 15 min.

### Cut-off value, repeatability, specificity and sensitivity of CRISPR-Cas12a based assay

**Fig. 4:**
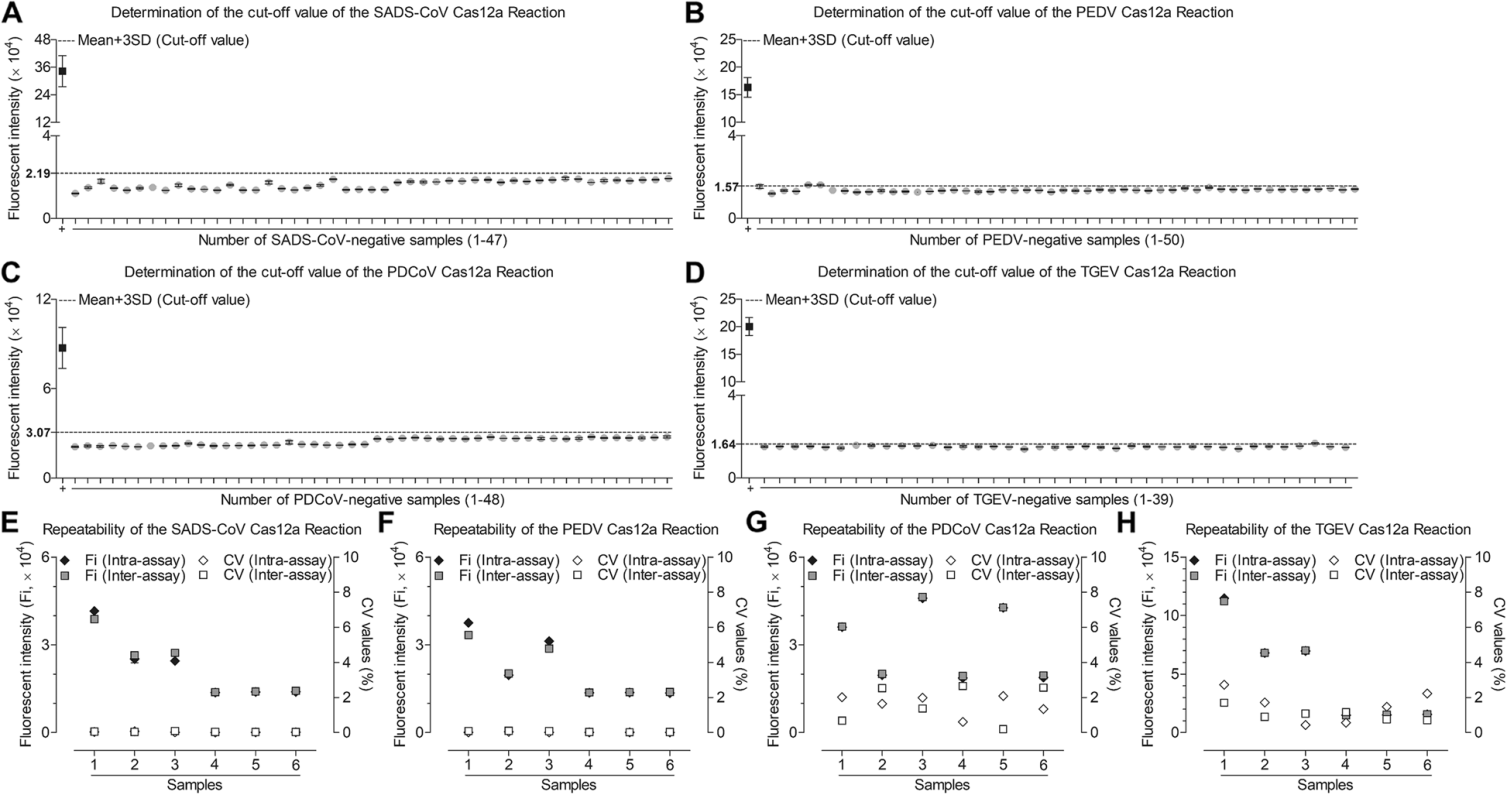
Determination of the cut-off value and repeatability of CRISPR-Cas12a assay. **A** 47 SADS-CoV-negative samples were tested using the CRISPR-Cas12a assay. **B** 50 PEDV-negative samples were tested using the CRISPR-Cas12a assay. **C** 48 PDCoV-negative samples were tested using the CRISPR-Cas12a assay. **D** 39 TGEV-negative samples were tested using the CRISPR-Cas12a assay. The mean fluorescence value of negative samples plus three SDs was used to calculated the cut-off value. **E-H** Six control samples (3 RT-qPCR-positive samples and 3 RT-qPCR-negative samples) were tested using the CRISPR-Cas12a assay and the fluorescence values of samples were used to calculate the CV to determine the intra- and inter-assay reproducibility. The data represents three independent experiments. The data is represented by mean ± SD, with *n* = 3

**Fig. 5:**
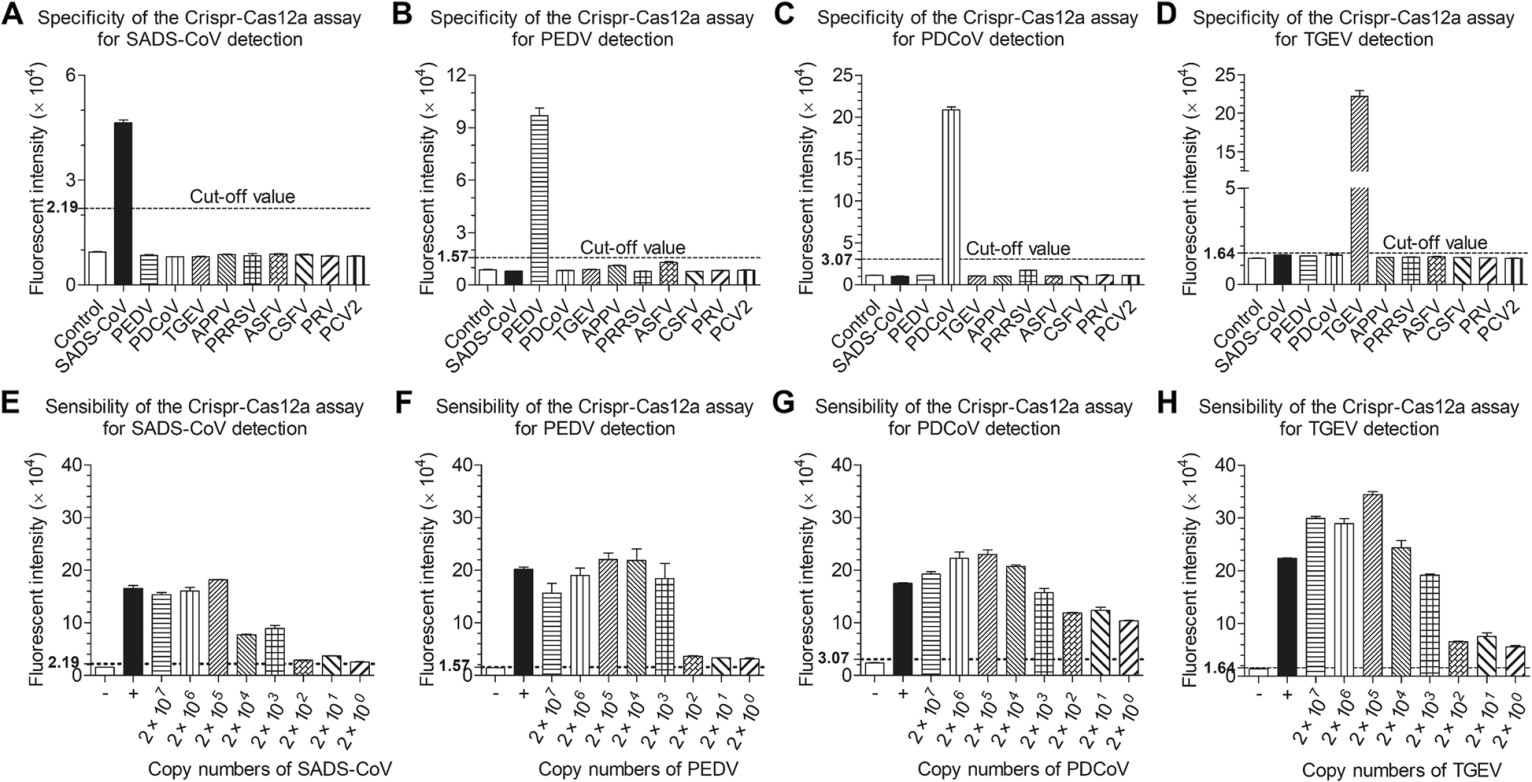
Determination of the specificity and sensibility of CRISPR-Cas12a assay. **A-D** SADS-CoV, PEDV, PDCoV, TGEV, APPV, PRRSV, ASFV, CSFV, PRV, PCV2 were tested using the CRISPR-Cas12a assay and the mean fluorescence value was calculated to determine whether the sample was positive or negative based on cut-off value. Virus was subjected to serial (log 10) and tested by CRISPR-Cas12a assay, ddH_2_O as the negative control.** E** Sensibility of CRISPR-Cas12a based assay in the detection SADS-CoV. **F** Sensibility of CRISPR-Cas12a based assay in the detection PEDV.** G** Sensibility of CRISPR-Cas12a based assay in the detection PDCoV. **H** Sensibility of CRISPR-Cas12a based assay in the detection TGEV The data represents three independent experiments. The data is represented by mean ± SD, with *n* = 3

As shown in Fig. 4A, the mean fluorescence intensity value of 47 SADS-CoV negative samples was 16,439.5 RFU, and the SD of these samples was 2101.1 RFU. Therefore, the cut-off value of SADS-CoV CRISPR-Cas12a based assay was calculated to be 2.19 × 10^4^ RFU. Similarly, the cut-off values of PEDV, PDCoV, TGEV CRISPR-Cas12a based assay were calculated to be 1.57 × 10^4^ RFU_,_ 3.07 × 10^4^ RFU_,_ 1.64 × 10^4^ RFU (Fig. 4B-D). The intra- and inter-assay CV of the CRISPR-Cas12a based assay were all determined to be less than 10% (Fig. 4 E–H). To determine the specificity of the CRISPR-Cas12a based assay for the four SECoVs, several common swine viruses were tested. As shown in Fig. 5A, the average fluorescence values of PEDV, PDCoV, TGEV, APPV, PRRSV, ASFV, CSFV, PRV, PCV2 were 0.850833 × 10^4^ RFU, 0.804633 × 10^4^ RFU, 0.812933 × 10^4^ RFU, 0.867267 × 10^4^ RFU, 0.8408 × 10^4^ RFU, 0.882933 × 10^4^ RFU, 0.8637 × 10^4^ RFU, 0.82633 × 10^4^ RFU and 0.822567 × 10^4^ RFU, respectively. All these values are smaller than 2.19 × 10^4^ RFU, suggesting that these viruses were negative by SADS-CoV CRISPR-Cas 12a based assay. Similarly, the CRISPR-Cas12a based assay for PEDV, PDCoV, TGEV exhibited high specificity (Fig. 5B-D). As shown in Fig. 5E-H, all the template concentration above 2 × 10^0^ copies/μL were identified as positive, indicating that the CRISPR-Cas12a based assays exhibited a limit of detection of 2 copies for the four SECoVs.

### Comparison of field samples detection between CRISPR-Cas12a based assay and RT-qPCR

**Fig. 6:**
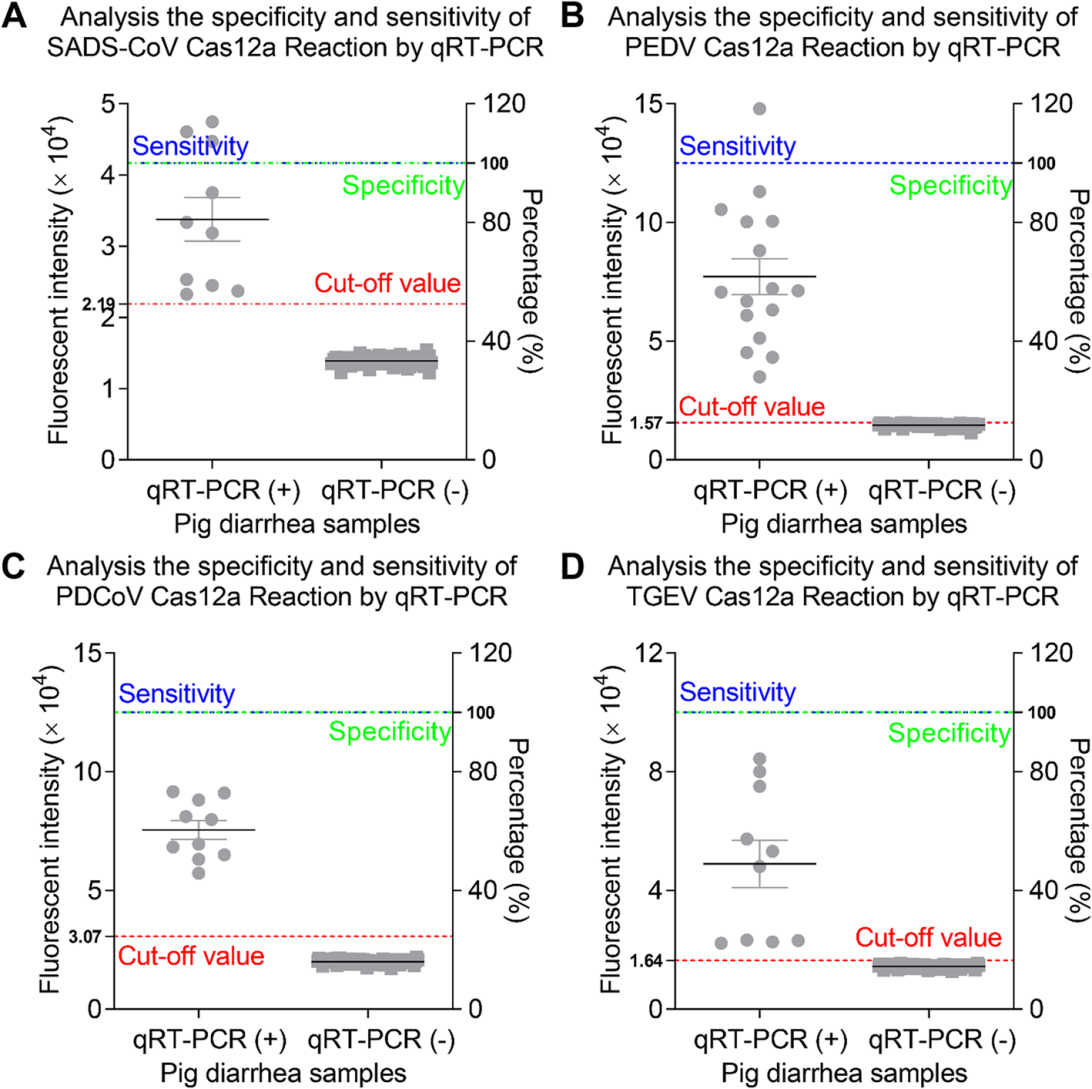
Validation of the CRISPR-Cas12a assay by RT-qPCR. A total of 106 pig diarrhea samples were examined by RT-qPCR. **A-D** The specificity and sensitivity of the CRISPR-Cas12a assay for SADS-CoV, PEDV, PDCoV and TGEV were analyzed based on the results of RT-qPCR. The sensitivity means consistency of positive samples detection, the specificity means consistency of negative samples detection

A total of 116 pig swab samples were tested by RT-qPCR and CRISPR-Cas12a assay, respectively. As shown in Fig. 6, the detection results of the CRISPR-Cas12a based assay for the four SECoVs had 100% agreement with RT-qPCR.

### Detection and analysis of SADS-CoV, PDCoV, TGEV and PEDV in field samples

**Fig. 7:**
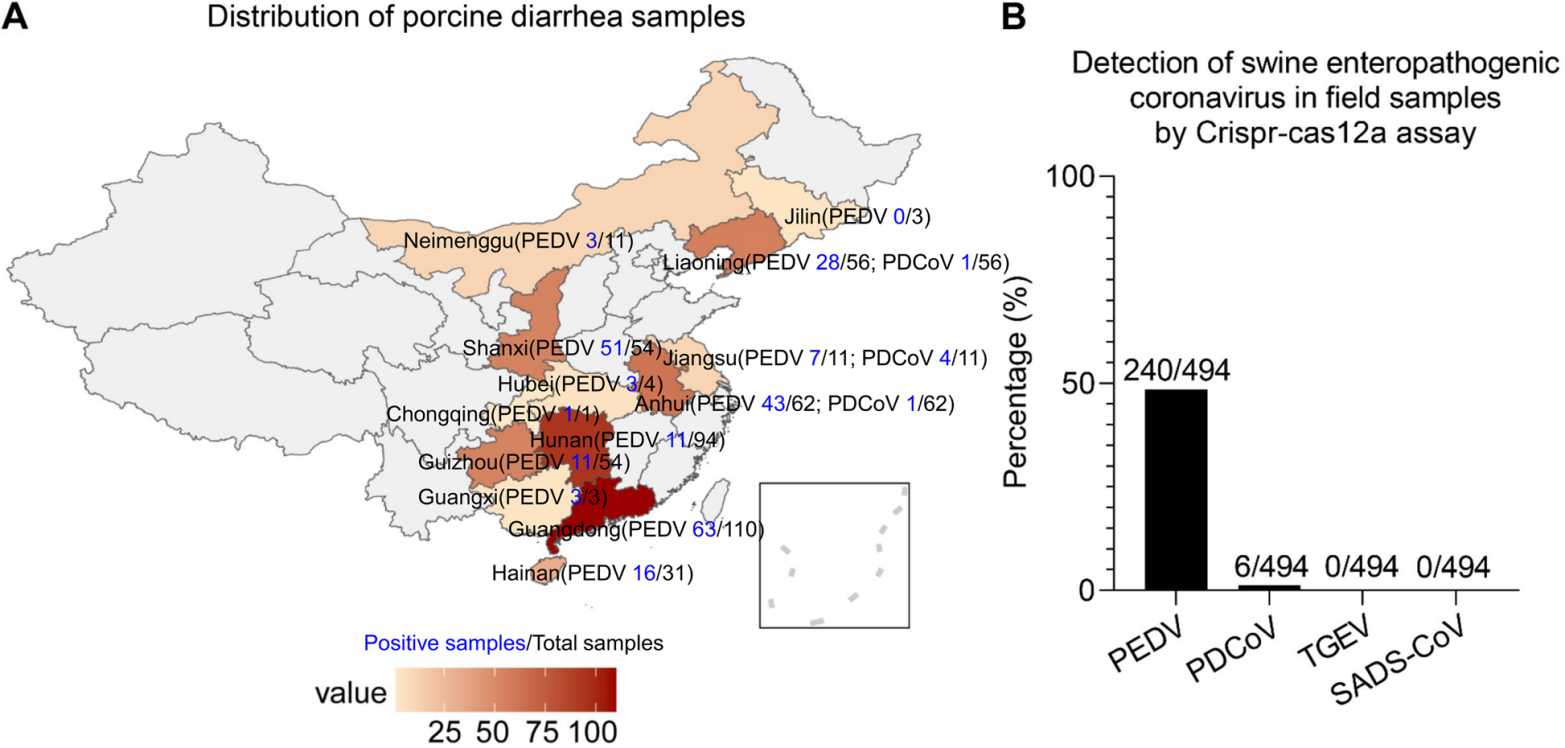
Rate of detection of PEDV, SADS-CoV, PDCoV and TGEV in pig diarrhea samples. A total of 494 pig diarrhea samples were collected from 55 large-scale pig farms in 13 provinces and cities (Guangdong, Guangxi, Guizhou, Hainan, Hubei, Hunan, Jilin, Jiangsu, Liaoning, Neimenggu, Anhui, Shanxi, Chongqing) in China from December 2021 to March 2023, and tested for presence of PEDV, SADS-CoV, PDCoV, TGEV by CRISPR-Cas12a assay. **A** The spatial distribution map of the pig diarrhea samples; (**B**) Rate of detection of PEDV, SADS-CoV, PDCoV and TGEV in pig diarrhea samples

To assess the positivity rate of SECoVs in the pig diarrhea samples across China, a total of 494 suckling pig diarrhea samples were collected from 55 large-scale pig farms in 13 provinces or municipal cities from December 2021 to March 2023, and the geographical distribution of samples was shown in Fig. 7A. All samples were detected by CRISPR-Cas12a based assay. As shown in Fig. 7B, 48.58% (240/494) of pig diarrhea sample were positive for PEDV and 1.21% were positive for PDCoV (6/494). Interestingly, SADS-CoV and TGEV were not detected.

## Discussion

Coronaviruses (CoVs) poses a significant and concerning risk to both animal and human health (Cui et al. 2019). SECoVs not only induce diarrhea in pigs across various age groups, leading to significant economic losses, but also possess a threat to public health because of the potential for cross-species transmission (Xu et al. 2019; Yang et al. 2019). Rapid, accurate, and practical detection methods play a crucial role in the monitoring and control of SECoVs, aiding in the prevention and management of disease outbreaks (Jung et al. 2020; Yan et al. 2022). In this study, a CRISPR-Cas12a based assay was developed and utilized to investigate the positivity rate of SECoVs in the diarrheic pig samplesof China.

CRISPR-Cas12a as a defense system for bacteria, combined with isothermal amplification for atom molar sensitivity and single base mismatch specificity, is widely used in medical diagnostics, environmental monitoring, and food pathogen detection (Bandyopadhyay et al. 2020; Shi et al. 2021). Notably, the CRISPR-Cas system has demonstrated its potential for rapid detection of SARS-CoV-2, as evidenced by numerous experimental results. This method has been shown to be safe, rapid, simple, and cost-effective approaches for virus detection (Liang et al. 2021; Mao et al. 2022; Sun et al. 2021; Xiong et al. 2020). Furthermore, it is known for low single base mismatch rate, and minimal instrument requirements compared to other detection methods (Gong et al. 2021; Wu et al. 2023). These studies prompted us to test whether CRISPR-Cas system can also be utilized for the detection of SECoVs. The selection of target genes affects the specificity and sensitivity of the assay. The *N* gene exhibits a remarkable degree of conservation during the genetic evolution of coronaviruses (He et al. 2020; Liu et al. 2021). Therefore, *N* gene was chosen as the detection target. High-score crRNAs for *N* genes were designed and the best crRNA were screened by Cas12a reaction. In order to accurately amplify a large number of target gene fragments in a short period of time, we chose RT-RPA as the amplification technique. This method exhibitted several adantages, including user-friendly operation, rapid amplification, and minimal instrument requirement (Tan et al. 2022). Since oligonucleotide sequences are essential for rapid and sensitive RPA, primers need to be screened (Xiong et al. 2020). We designed and screened the optimal performing primer pairs for amplification. We further established a CRISPR-Cas12a method for the detection of SECoVs by threshold determination, repeatability, specificity analysis, and analytical sensitivity. RT-qPCR is a commonly used detection method for viruses (Mackay et al. 2002; Pan et al. 2020). Our findings demonstrated that the CRISPR-Cas12a based assay yielded identical results to those obtained from RT-qPCR tests, indicating that the CRISPR-Cas12a assay was able to accurately detect SECoVs in pig diarrhea samples.

Piglet diarrhea can be attributed to various factors, such as viral, bacterial, parasitic, nutritional, environmental, and other influences. Among these, viral infections are frequently observed. PEDV, TGEV, PDCoV and SADS-CoV are major viral pathogens known to cause diarrhea in piglets (Li et al. 2022; Zhai et al. 2016). Based on the CRISPR-Cas12a based assay established in this study, PEDV (48.58%) was found to be the main pathogen causing suckling pig diarrhea in China in recent years, which was consistent with other studies (Li et al. 2023, Zhang et al. 2023). While only 1.21% of PDCoV has been detected and no TGEV or SADS-CoV have been identified, we still cannot rule out the possibility that these strains may still be present in pig farms across China. We need to expand the sample size and scope of testing in order to gain a comprehensive understanding of the prevalence of enteric coronavirus in pigs across China. The collective findings from this study provide compelling evidence that the CRISPR-Cas12a based assay developed for the detection of SECoVs is well characterized by its specificity, sensitivity, and repeatability suggesting a great potential of field application to detect SECoV infections. Nevertheless, some questions remain unanswered, necessitating further investigation. For instance, whether it is possible to specifically detect four SECoVs at the same time in a single Cas12a reaction? Elucidation of this will help us to develop a multiplex CRISPR-Cas12a based assay to detect all major SECoVs infection.

In conclusion, a CRISPR-Cas12a based assay for SECoVs with high specificity, sensitivity and good repeatability was established in this study, which could facilitate large-scale detection of SECoVs in the pig farms.

## Data Availability

All data supporting the findings of this study are available from the corresponding author upon request.
